# Observation of elliptically polarized light from total internal reflection in bubbles

**DOI:** 10.1038/s41598-020-65410-5

**Published:** 2020-05-26

**Authors:** Sawyer Miller, Yitian Ding, Linan Jiang, Xingzhou Tu, Stanley Pau

**Affiliations:** James C. Wyant College of Optical Sciences, University of Arizona, Tucson, AZ 85721 USA

**Keywords:** Applied optics, Optical physics, Optics and photonics

## Abstract

Bubbles are ubiquitous in the natural environment, where different substances and phases of the same substance forms globules due to differences in pressure and surface tension. Total internal reflection occurs at the interface of a bubble, where light travels from the higher refractive index material outside a bubble to the lower index material inside a bubble at appropriate angles of incidence, which can lead to a phase shift in the reflected light. Linearly polarized skylight can be converted to elliptically polarized light with efficiency up to 53% by single scattering from the water-air interface. Total internal reflection from air bubble in water is one of the few sources of elliptical polarization in the natural world. Stationary and dynamic scenes of air bubbles in water in both indoor and outdoor settings are studied using an imaging polarimeter. Our results are important for studies in fluid dynamics, remote sensing, and polarimetry.

## Introduction

Circularly and elliptically polarized light is atypical in nature. Such sources can occur from interaction with chiral materials^1–4^, reflection from materials with a non-zero imaginary index of refraction (i.e. metals)^5,6^, and total internal reflection (TIR)^5,6^. Chiral materials in nature are often associated with biologically active molecules such as naturally occurring amino acids and sugars^7,8^. Reflection from metals usually indicates a man-made object in the natural world^9^. TIR in the natural world most commonly occurs at a water-air interface, where incident linearly polarized light undergoes a phase shift to become elliptically polarized. Studies have been performed showing the utility of underwater imaging using circular and elliptically polarized light^10^ as well as the observation of elliptical polarization vision in animals^11–13^. Notably, this includes the Mantis shrimp^14^ and other crustaceans. Other studies have shown the presence of circular polarization in bioluminescence^15^.

Air bubbles in various media are abundant in everyday life. Common examples include agitated water in waterfalls and streams, gas trapped in liquid and ice, and boiling water. Studies have shown that the number of bubbles or whitecaps in the ocean have a significant impact on the climate^16,17^. When travelling through water, a naval craft produces a wake, a source of air-water mixing, which has been tracked using synthetic aperture radar^18^. Other studies have shown the use of bubbles in medicine for contrast agents^19^. Finally, bubbles are also used for indicating tectonic hazards in geology^20^.

In this work, the polarization state of light after a total internal reflection from a bubble is studied in both static and dynamic settings. The TIR interface in question is the bubble’s water-air boundary. Our study shows a distinct elliptically polarized signature from the bubble after a TIR event when illuminated with linearly polarized light^21^. Sources of linearly polarized light used in this study include lasers and natural skylight.

## Theory

The Mueller-Stokes calculus is used to describe any polarized polychromatic light^5^. Here, the polarization state of light is described using a four component vector known as the Stokes vector $\overrightarrow{S}={[{S}_{0},{S}_{1},{S}_{2},{S}_{3}]}^{T}$. The properties of the Stokes vector can be simplified to quantities such as the degree of linear polarization (DoLP), angle of linear polarization (AoLP) or *ϕ*, and degree of circular polarization (DoCP). These are defined below:

1 $$\begin{array}{ccc}{\rm{DoLP}} & = & \frac{\sqrt{{S}_{1}^{2}+{S}_{2}^{2}}}{{S}_{0}}\\ {\rm{AoLP}} & = & \frac{1}{2}{\tan }^{-1}\,\left(\frac{{S}_{2}}{{S}_{1}}\right)\\ {\rm{DoCP}} & = & \frac{{S}_{3}}{{S}_{0}}\end{array}$$

Changes in the polarization state are calculated through multiplication with Mueller matrices. The Mueller matrix is a 4 × 4 matrix, which contains all the polarization properties of an optical object, including diattenuation, retardance, and depolarization. Multiplication of the Stokes vector by the Mueller matrix changes the polarization state of the incident light and describes the interaction of light with the polarizing object. In this work, we concentrate on the study of total internal reflection (TIR). TIR occurs at a boundary between two transparent dielectric materials where the angle of incidence is greater than the critical angle^6^, *θ*_*c*_.

2 $${\theta }_{c}={\sin }^{-1}\left(\frac{{n}_{2}}{{n}_{1}}\right)$$

In Eq. (2), *n*_1_ and *n*_2_ are the indices of refraction for the incident and refracted materials respectively. For the interface between water and air, the critical angle is calculated to be approximately 48°.

An arbitrary linear polarization state of light can be represented by a Stokes vector^10^: $\overrightarrow{S}={S}_{0}{[1,\cos 2\phi ,\sin 2\phi ,0]}^{T}$, where *S*_0_ is the total irradiance, and *ϕ* is the orientation of the electric field or AoLP. The Mueller matrix for TIR^22^ is given by Eq. (3):

3 $$M=[\begin{array}{cccc}1 & 0 & 0 & 0\\ 0 & 1 & 0 & 0\\ 0 & 0 & \cos \,\delta  & -\,\sin \,\delta \\ 0 & 0 & \sin \,\delta  & \cos \,\delta \end{array}]\,,\,{\rm{w}}{\rm{h}}{\rm{e}}{\rm{r}}{\rm{e}}\,\,\delta =2{\tan }^{-1}\left(\frac{\sqrt{{n}^{2}{\sin }^{2}\,\theta -1}}{n\,\cos \,\theta }\right)-2{\tan }^{-1}\left(\frac{n\sqrt{{n}^{2}{\sin }^{2}\,\theta -1}}{\cos \,\theta }\right)$$

Here, *θ* is the angle of incidence from water to air at the water-air interface and, *n* = 1.33, the index of refraction for water. Multiplying the incident Stokes vector by the Mueller matrix defined in Eq. (3), an output Stokes vector as a function of *ϕ* and *δ* is found. An efficiency can now be calculated as the ratio of the reflected DoCP to the incident DoLP^10^. The relation is shown below in Eq. (4).

4 $$\eta =\frac{{S}_{0}\,{\rm{DoCP}}}{{S}_{0}\,{\rm{DoLP}}}=\frac{|\,\sin \,\delta |\,|\,\tan \,2\phi |}{\sqrt{1+{\tan }^{2}\,2\phi }}$$

**Figure 1:**
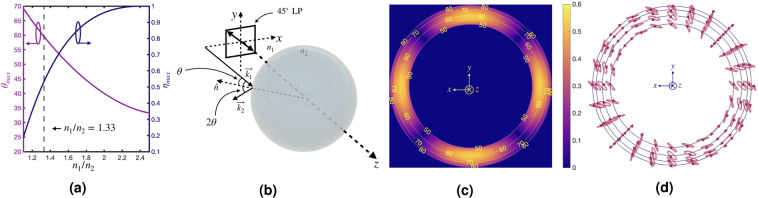
(**a**) A plot of maximum conversion efficiency (*η*_*max*_) from linearly to elliptically polarized light is shown as a function of the ratio of refraction indices. The angle of incidence (*θ*_*max*_) at which the maximum conversion efficiency occurs is also plotted as a function of the ratio of the refraction indices. The vertical line at *n*_1_/*n*_2_ = 1.33 represents water to air. (**b**) A schematic of the experimental setup is shown. An air bubble is suspended and then illuminated with 45° linearly polarized light, which has direction of ${\overrightarrow{k}}_{1}$ and is both incident to the bubble and reflected by TIR. The elliptically polarized light now travels in the direction of ${\overrightarrow{k}}_{2}$. (**c**) Contour plot of *η*_*max*_ as a function of incident angle *θ* for a single bubble is shown. 45° linear polarization is incident, and the plot peaks as expected near 60° angle of incidence. There is a cross-pattern, *η* = 0, where no conversion occurs as this corresponds to the local *s-* and *p-*polarization orientations. (**d**) Ellipses representing the elliptical polarization state of the light after undergoing TIR with 45° linear polarization incident are shown. The blue contour lines correspond to the angle of incidence in (**c**). The most elliptical polarization states are near the 60° angle of incidence.

The absolute values are taken of the two arguments in the numerator, as a positive efficiency is required. It is found the maximum conversion efficiency is 0.53 at an angle of incidence of 60.1° with an AoLP of 45°. Figure 1(a) shows how the efficiency, *η*_*max*_, changes as a ratio of the indices of refraction. The curve approaches unity as the ratio grows. Also plotted is the angle of incidence, *θ*_*max*_, at which the maximum conversion occurs. As the ratio of the indices of refraction grows, the angle becomes shallower. A vertical line, plotted at *n*_1_/*n*_2_ = 1.33, represents the ratio of water to air. A diagram of the coordinate system is shown in Fig. 1(b), where 45° linearly polarized light travels parallel to the *z*-axis with *k*-vector equal to ${\overrightarrow{k}}_{1}$. ${\overrightarrow{k}}_{1}$ defines an angle *θ* with the surface normal of the local bubble curvature, $\hat{n}$. The light experiences TIR and is reflected with a new *k*-vector equal to ${\overrightarrow{k}}_{2}$. In this diagram *n*_1_ is equal to 1.33, the index of refraction of water, and *n*_2_ is equal to one.

It is instructive to look at the distribution of conversion efficiency, as defined in Eq. (4), over the surface of a bubble. For simplicity, we assume the bubble to be perfectly spherical. Results are shown in Fig. 1(c), where the incident light AoLP = 45°. Each of the contour lines represents a different angle of incidence (*θ*) to the surface normal of the local curvature of the bubble. The local coordinates are referenced at the center of the figure to show the connection with Fig. 1(b). An efficiency of zero is recorded for those incident angles less than 48°, i.e. the critical angle for TIR between water and air. The cross-pattern in Fig. 1(c) is caused by the 45° linear polarization corresponding to local *s-* and *p-*polarizations, so no phase shift occurs, and the efficiency is zero. Figure 1(d) shows the resulting elliptical polarization state of the light after TIR as a function of position on the bubble. The highest ellipticity corresponds to the same locations in Fig. 1(c), where the efficiency is the highest. Notice that the cross-pattern is also formed where the incident 45° linear polarization corresponds to the local *s-* and *p-*polarizations. No phase shift occurs at these locations, and the reflected light remains linear, regardless of angle of incidence. Supplemental Video S1 shows how the pattern found in Fig. 1(d) rotates as the AoLP of incident light changes from 0°-180°.

The bubbles used in this work are formed in one of two ways, depending on conditions. Sets of data were taken with a stationary air bubble formed within a bulk of water. In this scenario, the formation of a spherical bubble is dictated by the surface tension of the water and Laplace pressure placed on the air bubble^23^. This relationship is shown below in Eq. (5), where *γ* is the surface tension, *R* is the radius of the bubble, and the quantity *P*_*i*_ − *P*_*o*_ is the difference between inside and outside pressure or the Laplace pressure.

5 $${P}_{i}-{P}_{o}=\frac{2\gamma }{R}$$

Moreover, other sets of data are taken with a continuous stream of water drops free-falling in air. Shearing effects as the water drops move in the air results in Kelvin-Helmholtz instability, thus mixing of air and water^24,25^. These mixing zones, or eddies, are where pockets of air are formed, emulating the air bubble in water interface.

## Experiments

**Figure 2:**
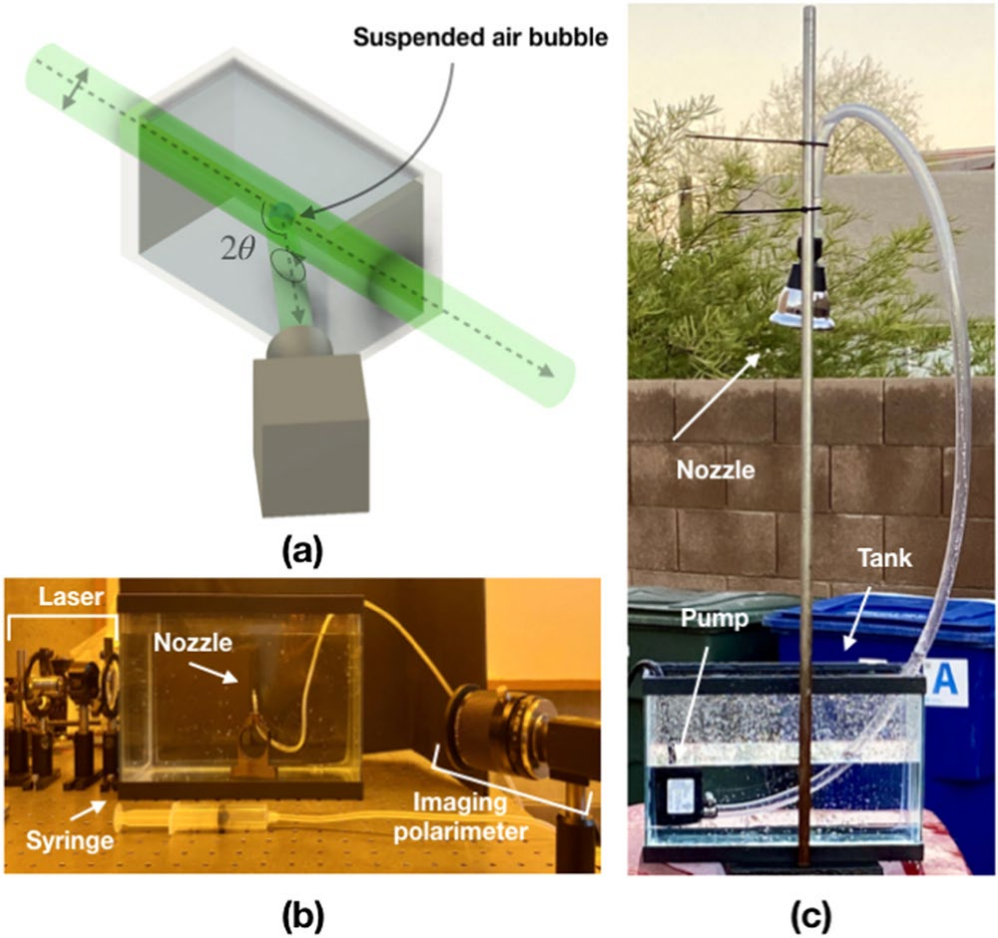
(**a**) Experimental setup showing the placement of the bubble inside the small tank filled with water. The bubble is illuminated by 45° linear polarization from a collimated 532 nm diode laser. The imaging polarimeter is placed at an angle 2*θ*. Not shown is the refraction angle out of the tank into the air, which is considered in the data collection. (**b**) Setup for static experiment is shown. The laser along with filtering and collimating optics are shown on the left; the syringe and nozzle are shown in the center. The imaging polarimeter along with optics and quarter waveplate are shown on the right. (**c**) Setup for dynamic experiment is shown. A shower head is used as a nozzle to create a stream of water drops down to the tank. A small pump is used to circulate the water. The imaging polarimeter is out of view but situated directly below the tank pointed upward.

Three different light sources with different DoLP are used in this study. One source is the diode laser, which emits light of linear polarization. Another source is natural skylight, which is partially linearly polarized with maximum theoretical DoLP ≈ 0.75–0.80, caused by single Rayleigh scattering^26,27^. The third source is a fluorescent lamp, which outputs unpolarized light and serves as a comparison to linearly polarized sources. The DoLP of the sources ranges from unity (diode laser), to 0.70 (natural skylight), down to 0 (fluorescent light). The indoor experimental setup is shown in Fig. 2(a,b). The outdoor experimental setup is shown in Fig. 2(c). An imaging polarimeter is utilized to measure the scattered light in both setups.

### Static scenes

#### Indoor environment

A small aquarium with glass walls was filled with water, and a nozzle was placed in the tank. The nozzle, connected to a tube and syringe, allowed control over the bubble to be formed inside the tank. Multiple bubbles could be inserted into the tank with the syringe and nozzle.

**Figure 3:**
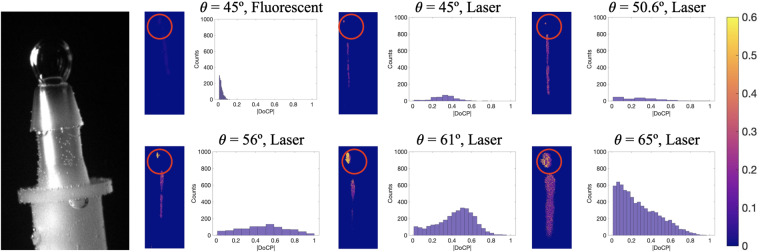
Image of single air bubble in water on top of nozzle is shown on the left. Sets of |DoCP| images for different illuminations and angle of incidence are also shown. For each |DoCP| image, there is a corresponding histogram showing the |DoCP| pixel counts in each image. The red circle denotes the area of interest, where the bubble resides and the data for the histogram. All readings of zero are not included, and all data is plotted on the same scale.

A stationary bubble, shown in Fig. 3, was formed on the tip of the nozzle. Fluorescent lamp and collimated laser were utilized for illumination in the indoor experiments. For laser illumination, the angle between the imaging polarimeter and the direction of the laser, along with the angle of incidence to the bubble, are varied from 45° to 65°. Due to the refraction out of the tank into the air and the physical restrictions between the aquarium and the imaging polarimeter, angles greater than 65° cannot be imaged. According to Fig. 1(c), no DoCP should be observed below 48° angle of incidence, the critical angle. Our results are summarized in Fig. 3.

The imaging results of the stationary bubble can be explained in the context of our theoretical analysis. The source used in the fluorescent illumination was not collimated, nor was it polarized. Without a collimated source, the angle of incidence to the bubble cannot be accurately determined. Therefore, many angles of incidence are recorded at the *θ* = 45° position. Additionally, without incident light that is polarized, the reflected light will remain unpolarized as the conversion efficiency is zero. However, referring to Fig. 3, small amounts of |DoCP| are measured and shown in the histogram due to interpolation error and the varying angles of incidence. The fluorescent source was setup in the same position as the laser, so the illumination came from the same direction. Data for the fluorescent example was taken with the camera in the *θ* = 45° position. The remaining examples are illuminated with laser light as depicted in Fig. 2(a). No TIR is expected below 48°. The figure showing incidence at 45°, shows near zero |DoCP| signatures. The value of |DoCP| peaks at 60°, and then starts to decline as expected as the angle of incidence increases. The measurement at 65° shows slight fringes presented in the |DoCP| image. These are intensity fringes, and a full discussion is presented in Supplemental Discussion 1. It is important to note that all histograms are plotted on the same scale to accurately represent the signal each angle of incidence produced for the imaging polarimeter.

#### Outdoor environment

**Figure 4:**
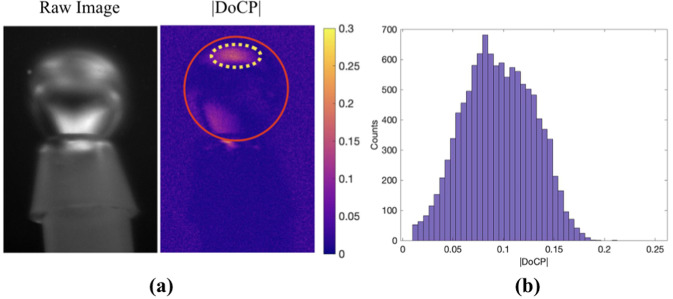
(**a**) Image shows a bubble illuminated by linearly polarized skylight. The same nozzle used in Fig. 3 is used in this setup. The |DoCP| image shows regions of elliptically polarized light from total internal reflections. The red circle denotes the interface of the air bubble in water. The yellow oval represents the area plotted in the histogram. (**b**) Histogram of the yellow encircled region in the |DoCP| image. Again, data of zero count is removed from the histogram for clarity.

Polarized light scattering of air bubbles in water was studied outdoors using natural skylight. Due to Rayleigh scattering, skylight has been shown to be highly linearly polarized, up to 75% in theory. The DoLP of the skylight varies with the position of the Sun, and highest DoLP is found in regions 90° away from the Sun. In our experiment, the measurements were taken at dawn, where the Sun was near the horizon. High DoLP is directly overhead at the zenith position^26^. The imaging polarimeter was positioned to be below the tank, looking up, but maintaining the 2*θ* angle between the bubble, incident, and reflected light. During the data collection, the DoLP was measured to be about 0.70 directly above at the zenith position. Additional information about the polarization of the surroundings is given in Supplemental Discussion 2. The experimental setup was rotated until the incident linear polarization from above was oriented at 45°. The raw image, |DoCP|, and histogram showing the |DoCP| pixel counts are presented in Fig. 4.

The |DoCP| histogram in Fig. 4(b) shows a range of values up to 0.20. The regions of highest specular reflection, near the top of the bubble, are areas where the incident linear polarization is being reflected. The maximum |DoCP| registered in the image is about 0.20, which is lower than the theoretical maximum of 0.37. Losses in signal could arise from nonuniform skylight illumination, interpolation error, and a non-ideal camera position where the 2*θ* camera position does not correspond to the *θ*_*max*_ of 60.1°.

### Dynamic scenes

Studies of moving air bubbles in water were performed in both indoor and outdoor environments. To accomplish this, a small pump was inserted to generate dynamic bubbles in the water. A second experiment setup was also arranged to pump water out of the tank to study a stream of irregular shaped water drops.

#### Indoor environment

Three different scenarios were considered in the indoor study. These include moving air bubbles in water, randomly shaped and sized water drops falling in air, and a smooth laminar flow of water stream in air. Each configuration was illuminated with both laser and fluorescent light.

**Figure 5:**
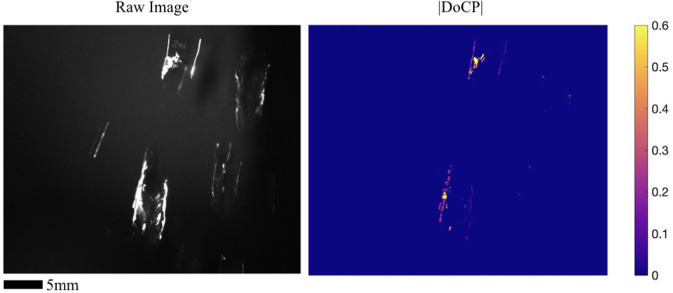
Indoor images of irregular shaped water drop illuminated by collimated laser light. High |DoCP| is recorded where the local geometry of the water drops forces TIR to occur.

Supplemental Videos S2 and S3 show air bubbles moving in water illuminated by laser light and fluorescent light respectively. As expected, Supplemental Video S2 shows flashes of high |DoCP| when a bubble is illuminated by laser light when the camera is placed in the correct position to capture the reflected light from TIR. Flashes of |DoCP| near the theoretical maximum of 0.53 are recorded in the video. A single frame of Supplemental Video S2 is shown in Fig. 5. Supplemental Video S3 does not exhibit any |DoCP| other than random noise in the background due to low signal. This is expected as the fluorescent light is not polarized. The camera is placed at the 61–65° position of the static indoor scenes.

Supplemental Video S4 shows irregular, i.e. not spherical, drops of water falling in air through the laser illumination. Similar to Supplemental Video S2, S4 displays flashing |DoCP| values near the theoretical limit of 0.53, where the local geometry of the water drop serves as the local water-air interface to establish conditions resulting in TIR. The camera is positioned so the light captured is incident and reflected near the *θ*_*max*_ of 60°.

Finally, a continuous laminar flow of water in air was imaged with laser illumination, shown in Supplemental Video S5, and with fluorescent illumination, shown in Supplemental Video S6. In these cases, the water stream is surrounded by a smooth and continuous interface with the surrounding air. No air packets or eddies were present inside the water stream. The light sources were directed at the water stream perpendicularly to the flow direction. The angle of incidence from water to air is not high enough for TIR to occur. Therefore, no reflected elliptical polarization is expected and observed. The camera position is also placed near the theoretical *θ*_*max*_ of 60°.

#### Outdoor environment

**Figure 6:**
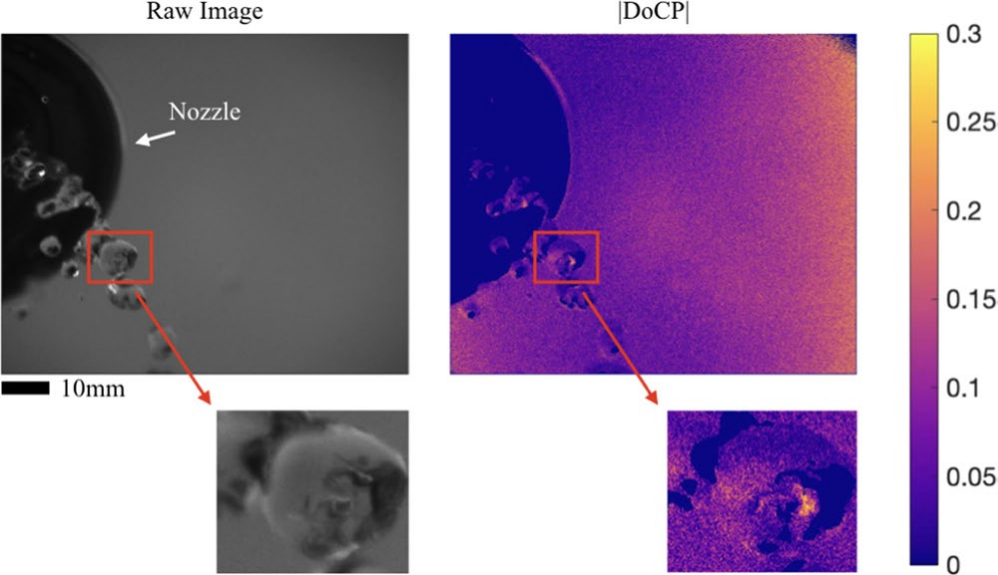
Outdoor images of an irregular shaped water drop illuminated by polarized skylight coming out of a nozzle are shown. High |DoCP| is recorded. Magnified views show details of the water drop.

The setup from the outdoor static scene was modified by adding a water pump to generate irregular shaped water drops as shown in Fig. 2(c). The data was taken at dusk. The DoLP of the sky was located at the zenith point and has a value close to 0.70. Additional information describing the polarization of the surroundings is given in Supplemental Discussion 2. The setup was rotated relative to the AoLP of the incident skylight, so the skylight was 45° linearly polarized incident to the bubble. Supplemental Video S7 shows the raw image and |DoCP| changing as the irregular drops fall from the nozzle. The theoretical |DoCP| was estimated to be 0.37; however, the polarimeter recorded maximum values between 0.20 and 0.25. Sources of deviation include nonuniform skylight illumination, non-ideal camera position where the *θ*_*max*_ position of 60° was not met, and interpolation error. A single frame from Supplemental Video S7 is shown below in Fig. 6.

Images in Fig. 6 show a constant stream of water drops flowing out of a nozzle in air. Bag-formation features, commonly seen in falling water drops, are observed where a volume of air is surrounded by a bag- or a rim-like water shell^28,29^. It is believed that these features provide water-air interfaces locally and transiently satisfy the TIR conditions. Therefore, hot spots of |DoCP| were observed at those locations of TIR of polarized skylight. A magnified view shows areas of high |DoCP|, where air is surrounded by water shell in a drop

## Discussion

Both static and dynamic scenes of air bubble in water show well-defined signatures of reflected elliptically polarized light. Nevertheless, several caveats must be taken into consideration for accurate detection and observation of elliptical polarized light in nature.

First, as stated in the theory section and in experimentation, the maximum |DoCP| which can be measured from a single reflection is 53% for incident light that is purely linearly polarized. Thus, partially polarized incident light will lead to reflected light of |DoCP| lower than the maximum. Second, the camera must be placed in the correct position relative to the incident AoLP and the bubble for observation of high |DoCP | . Referring back to Fig. 1(c), as the AoLP changes, the cross-pattern rotates; consequently, the camera position is of upmost importance to capture the maximum signal. In dynamic cases, one observes flashes of |DoCP| when the travelling bubbles are in the correct positions passing through the illumination beam. Supplemental Discussion 3 provides a derivation for |DoCP| in case of illumination by all linear polarization states.

Another important factor to consider is bubble. As the density of the bubbles increases, the magnitude of the |DoCP| signal generally decreases. This is due to the multiple reflections at each bubble interface, which change the polarization state and the direction of propagation. Studies have shown a limit of 10^5^/m^3^ is where significant depolarization starts to begins^30^. This is evident in polarization returning LIDAR systems studying ocean and wave dynamics where a depolarization correction is needed to quantify oceanic whitecaps^31,32^.

Observing elliptically and circularly polarized light has applications in surveillance^33^ and metrology. It has been shown that circularly polarized light retains its polarization state as it travels through turbid media better than linear polarization states^34–38^. For remote imaging over long distances through smoke and haze, polarization imaging, without active illumination such as LIDAR^39^, may provide better signal-to-noise in comparison with conventional color or monochrome measurements. The magnitude of DoCP correlates with the surface shape of the bubble. Imaging of spatial distribution of scattered polarized light under control illumination can provide accurate shape and size information of bubbles in real time as well^40^.

In summary, elliptical polarization signatures were observed, in both an indoor laboratory setting and outdoor setting. Additionally, these signatures were captured in stationary scenes, as well as dynamic scenes. |DoCP| as a function of angle of incidence was recorded and matched closely to the developed TIR theory for the geometry of a spherical bubble. Maximum |DoCP| values of 0.53 were measured in both the static and dynamic scenes in the indoor setting. |DoCP| in the outdoor settings were observed, but were up to 50% lower than the theoretical maximum. This can be attributed to partial linear polarization of skylight and non-optimal camera placement.

## Methods

The imaging polarimeter used in this work was a monochrome Lucid Vision Labs Triton division of focal plane (DoFP) polarimeter. The detectors resolution is 2048 × 2448 pixels. This imaging polarimeter is only sensitive to linear polarization states. An achromatic quarter waveplate was added in front of the imaging lens to allow for measurement of elliptically polarized light and DoCP. Additional discussion is provided in Supplemental Discussion 3. The quarter waveplate, with an operating band from 400–900 nm, was manufactured by Boulder Vision Optik. Imaging lenses included a Computar 55 mm macro lens focused at infinity at *f*/2.8 with a 5X close-up lens added for the indoor experiments. The stationary outdoor experiment used a Computar 55 mm macro lens focused at infinity at *f*/2.8 with a 10X close-up lens. Additionally, a 10 nm bandpass filter, centered at 510 nm, was added to reduce noise. The dynamic outdoor scene used a Canon 102 mm lens focused at infinity at *f*/2.

A common issue of DoFP polarimeters is residual edge effects inherent in the data sampling and processing^41,42^. These artifacts are consequences of the fact that each pixel has a different instantaneous field of view from the adjacent pixels used in the data reduction process. An edge detection/bicubic interpolation algorithm^43^ was used for all images and video frames to mitigate the residual edge effects. Further discussion of the calibration and verification of the interpolation algorithm is given in Supplemental Discussion 4.

The laser used in the indoor experiments was a 532 nm laser diode rated at 30–50 mW of output power, corresponding to a varying input voltage of 3.0–3.7 V.

## Supplementary information

Supplementary Information.

Supplementary Video S1

Supplementary Video S2

Supplementary Video S3

Supplementary Video S4

Supplementary Video S5

Supplementary Video S6

Supplementary Video S7
