# Supplementary material for: Observation of elliptically polarized light from total internal reflection in bubbles
Source: Sci Rep. 2020 May 26;10:8725. doi: 10.1038/s41598-020-65410-5 (PMC7250915; doi:10.1038/s41598-020-65410-5)
Supplement: Supplementary file 1 — Supplementary Information. [file 41598_2020_65410_MOESM1_ESM.pdf]

Observation of elliptically polarized light from total internal reflection of bubbles: Supplemental Discussion

Sawyer Miller[1,+], Yitian Ding[+], Linan Jiang , Xingzhou Tu , and Stanley Pau[2]

James C. Wyant College of Optical Sciences, University of Arizona, Tucson, AZ, 85721, USA
[1]samiller@optics.arizona.edu
[2]spau@optics.arizona.edu
[+]these authors contributed equally to this work

Supplemental Discussion 1

Previous studies have shown that interference fringes can be observed when a bubble is illuminated with coherent laser light, and this information can be utilized to quantify the dimension and shape of the bubble[1]. The laser light that undergoes TIR at the water-air interface combines and interferes with the laser light that is reflected from the air-water interface inside the bubble to produce intensity fringes. In general, fringe is observed when the camera is defocused, allowing the two beams to overlap. In our study of polarized light scattering of water bubbles, we observe similar intensity fringes. Intensity image and |DoCP| image of a laser-illuminated water bubble are shown in Fig. S1. A red circle denotes the outline of the bubble, and the location of the nozzle is indicated as well. The image is out of focus, allowing the two reflected beams to interfere at the focal plane of the camera.

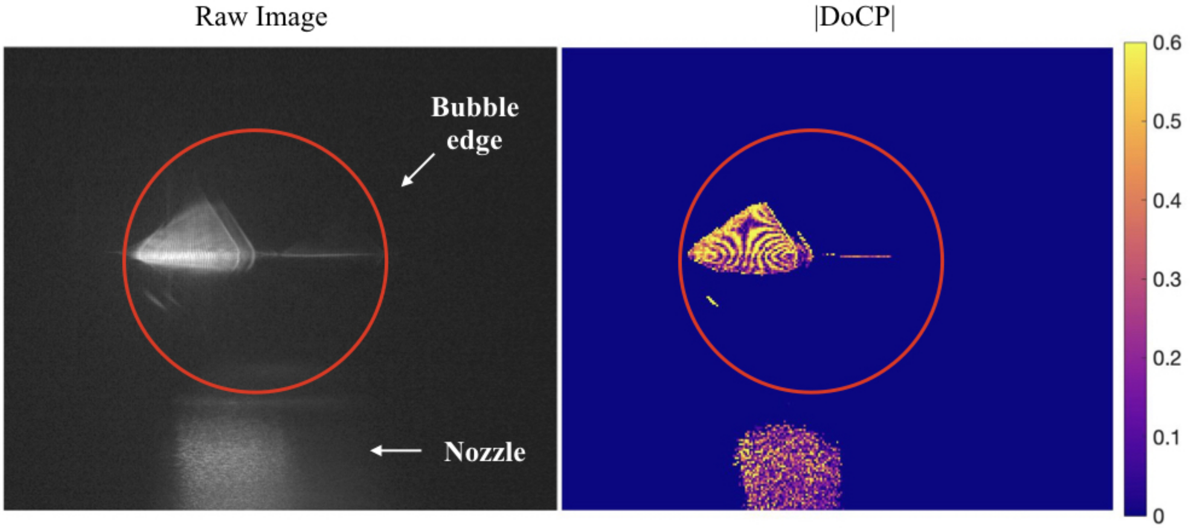

Figure S1. Intensity image of a defocused water bubble is shown on the left, and a |DoCP| image of the same bubble is shown on the right, revealing a multiple fringe pattern. The red circle denotes the location of the bubble's edge.

Supplemental Discussion 2

To quantify the direction and magnitude of the skylight's polarization state, we measured the sky's degree of linear polarization (DoLP) and angle of linear polarization (AoLP) using a fisheye lens. The experimental setup was subsequently rotated to allow the AoLP of the incident light to be at 45° relative to the bubble and the camera. It has been shown that the maximum DoLP from Rayleigh scattering is about 75%[2].

Outdoor static scene

Images of DoLP and AoLP of the sky taken at dawn are shown in Fig. S2. NA on the AoLP colorbar corresponds to areas in the DoLP image where the degree of polarization is too low to accurately determine the angle of polarization. For these images, the threshold was set to less than 5% DoLP.

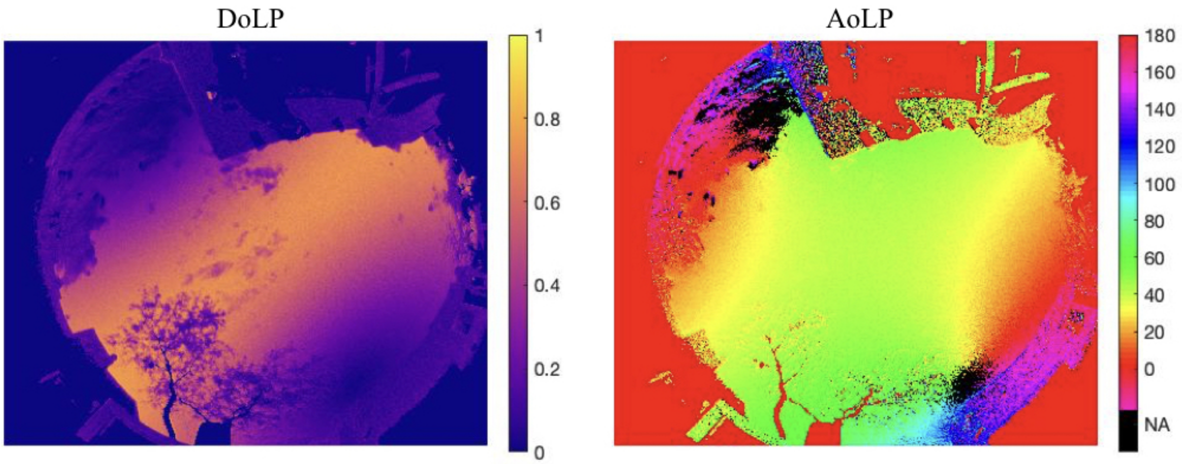

Figure S2. . Images of DoLP (left) and AoLP (right) of the sky were taken immediately before experiment on the static scene.

Outdoor dynamic scene

Images of DoLP and AoLP of the sky taken at dusk are shown in Fig. S3.

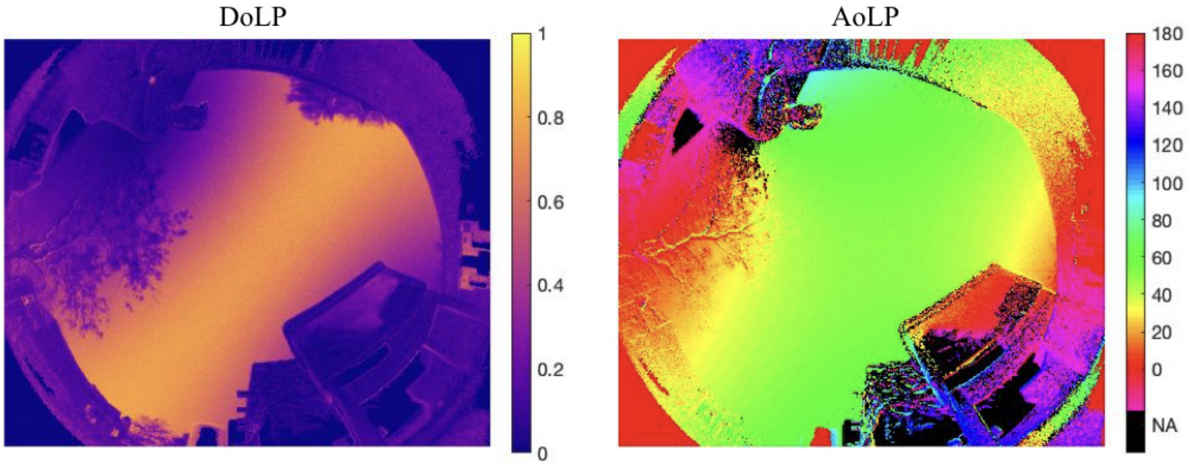

Figure S3. Images of DoLP (left) and AoLP (right) of the sky were taken immediately before experiment on the dynamic scene.

Supplemental Discussion 3

The camera used in the experiments is only sensitive to linear polarization states. For the camera to be sensitive to circular and elliptical polarization states, a quarter wave plate is added in front of the imaging lens. The quarter wave plate will rotate the plane of sensitivity from linear polarization states to circular and elliptical states. More precisely, in its initial configuration, the camera is more accurate in determining the degree of linear polarization (DoLP). With the quarter waveplate, the camera is now most accurate in determining the degree of circular polarization (DoCP). The Mueller-Stokes calculation is shown below.

$$M_{QWP} \cdot \vec{S}_0 = \vec{S}_1 \tag{S1}$$

where M_{QWP} is the Mueller matrix for the quarter waveplate oriented with the fast-axis at 90 degrees, and $\vec{S}_0$ and $\vec{S}_1$ are the input and output Stokes vectors respectively. Equation (S2) is expanded below to show how the camera becomes sensitive to the S_3 component of the incident Stokes vector.

$$\begin{bmatrix} 1 & 0 & 0 & 0 \\ 0 & 1 & 0 & 0 \\ 0 & 0 & 0 & -1 \\ 0 & 0 & 1 & 0 \end{bmatrix} \cdot \begin{bmatrix} S_0 \\ S_1 \\ S_2 \\ S_3 \end{bmatrix} = \begin{bmatrix} S_0 \\ S_1 \\ -S_3 \\ S_2 \end{bmatrix} \tag{S2}$$

The camera is now sensitive to the S_3 of the incident Stokes vector, and it can measure the DoCP of the incident light. However, no simultaneous measurement can occur where DoLP can be made, for this camera is not a full-Stokes polarimeter.

Supplemental Discussion 4

The Lucid Triton imaging polarimeter was first calibrated to ensure accurate measurement results. A collimated broadband source and a rotating wire grid polarizer were utilized for calibration. An image was taken at different polarizer angles to calculate the data reduction matrix[3]. Data without calibration was compared to data with calibration. It was found that data without calibration was very accurate for this camera. A test scene, comprising of a chopper wheel with the outer holes filled with linear polarizers and the inner holes filled with circular polarizers, was set up. The chopper wheel was illuminated with a broadband source from behind. Images of DoLP are shown in Fig. S4.

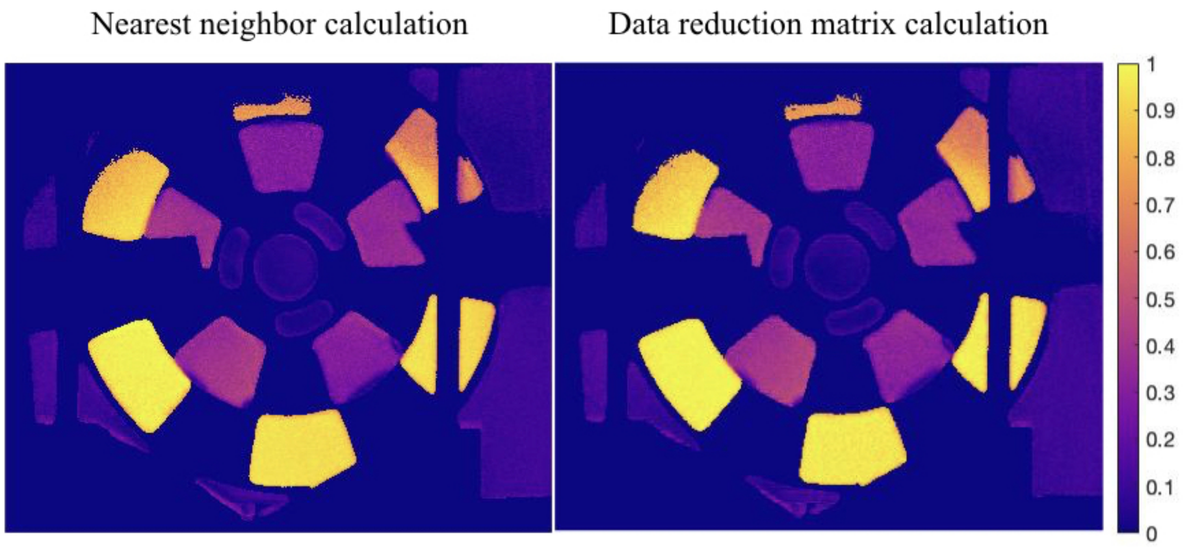

Figure S4. DoLP images of a chopper wheel are shown. The left image shows data with nearest neighbor calculation, and the right image shows data with data reduction matrix calculation. Both images are very similar to each other.

The two images in Fig. S4 are compared by subtracting one from the other. Ninety-four percent of the corresponding pixels across both images are within 5% pixel value. This calculation shows that the camera can provide accurate results without the need for a data reduction matrix analysis.

To mitigate the edge artifact problem common in degree of focal plane (DoFP) polarimeters, such as the Lucid Triton, an edge detection/bicubic interpolation algorithm was employed[4].

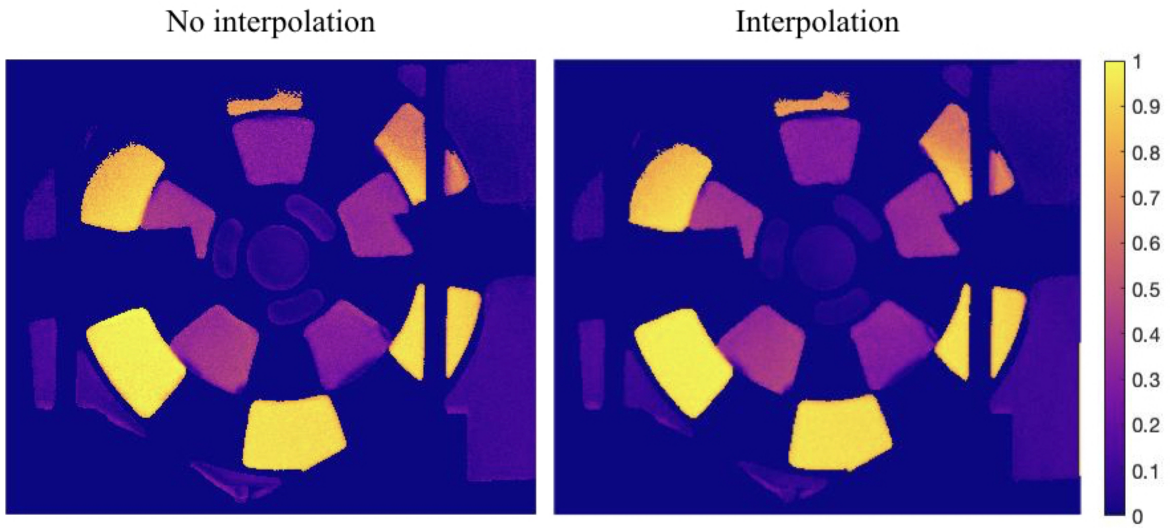

Figure S5. DoLP images with no interpolation (left) and interpolation (right) are shown. Comparison of the images demonstrates how the interpolation algorithm smooths the data.

Figure S5 shows the same chopper wheel as in Fig. S4. The DoLP is presented in each image, with no interpolation performed on the left, and interpolation performed on the right. The same calculation is shown in Fig. S6, but with the colormap saturated to show more detail in the image. Reduction of the edge artifacts from the interpolation algorithm is evident, especially near the center of the chopper wheel.

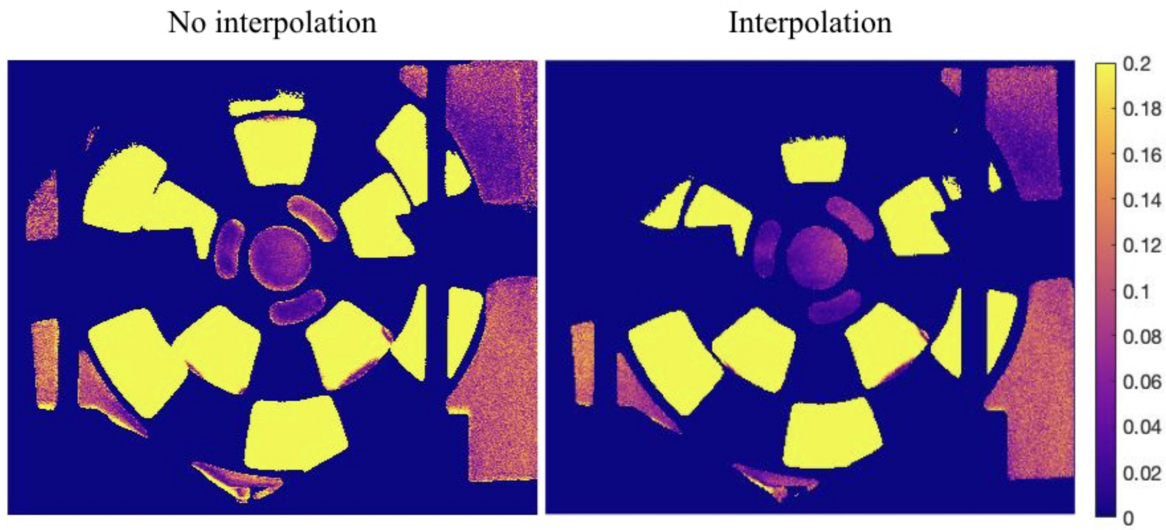

Figure S6. DoLP images for a set of saturated data with no interpolation (left) and with interpolation (right) are shown. The data is saturated to show the detail of the edge artifacts, especially near the center. The interpolation algorithm greatly reduces the edge artifacts.

Supplemental Discussion 5

Captions of all Supplemental Videos.

Supplemental Video S1: Ellipses representing the elliptical polarization state of the light after undergoing TIR with linear polarization incident are shown. The AoLP of the incident polarization is 0°-180°.

Supplemental Video S2: (Left) Raw video shows air bubbles moving dynamically through a tank of water. The bubbles are illuminated by a collimated laser. |DoCP| video of the moving bubbles is shown on the right. The camera is placed at an

angle that allows capturing the light that experiences TIR. Due to the laser illumination, |DoCP| values are recorded close to the theoretical maximum of 0.53.

Supplemental Video S3: (Left) Raw video shows air bubbles moving dynamically through a tank of water. The bubbles are illuminated by a non-collimated fluorescent source. |DoCP| video of the moving bubbles is shown on right. The camera is placed in the same position as in Supplemental Video S2. Due to the unpolarized nature of fluorescent light and zero valued conversion efficiency, low levels of |DoCP| are recorded.

Supplemental Video S4: (Left) Free-falling, non-spherical droplets of water are illuminated by collimated laser light and captured by the imaging polarimeter. |DoCP| video of the free-falling droplets of water is shown on the right. Due to the instability of the free-falling water, the water-air interface can approach angles found in the water-air bubble scenario. TIR is recorded, and values of |DoCP| approach the theoretical maximum of 0.53 due to laser illumination.

Supplemental Video S5: (Left) Raw video of a laminar flow of water being illuminated by collimated laser light. |DoCP| video of illuminated laminar flow is shown on the right. The laser illumination is coming from the left side of the frame and the light never experiences an interface with an angle of incidence near the critical angle. No TIR occurs, and little to no |DoCP| is recorded.

Supplemental Video S6: (Left) Raw video shows a laminar flow of water being illuminated by non-collimated fluorescent light. |DoCP| video of the laminar flow illuminated by fluorescent light is shown on the right. The fluorescent source is not polarized, and the angle of incidence for the light never approaches the critical angle at the water-air interface. No TIR will occur, and due to the unpolarized light source used, the conversion efficiency is zero. Little to no |DoCP| is observed.

Supplemental Video S7: (Left) Raw video shows non-spherical drops falling from a defocused nozzle in the background. The scene is illuminated by natural skylight. The camera is positioned to be looking up at the falling drops with the sky in the background. |DoCP| video of the falling drops is shown on the right. The instability in the falling droplet allows for the incident light to exceed the critical angle at the water-air interface, thus producing TIR. The DoLP of the skylight at the time of data capture was 0.70, and the AoLP of the incident light was 45°. The recorded |DoCP| was 0.20-0.25, which is lower than the theoretical maximum of 0.37.

References

1. Kawaguchi, T., Akasaka, Y. & Maeda, M. Size measurements of droplets and bubbles by advanced interferometric laser imaging technique. *Meas. Sci. Technol.* **13**, 308–316, DOI: 10.1088/0957-0233/13/3/312 (2002).
2. Konnen, G. P. *Polarized light in Nature* (Cambridge University Press, 1985).
3. Chipman, R., Lam, W. & Young, G. *Polarized Light and Optical Systems* (CRC, 2019).
4. Zhang, J., Luo, H., Hui, B. & Chang, Z. Image interpolation for division of focal plane polarimeters with intensity correlation. *Opt. Express* **24**, 20799–20807, DOI: 10.1364/OE.24.020799 (2016).
