# Supplementary figures and images for: Observation of elliptically polarized light from total internal reflection in bubbles
Source: Sci Rep. 2020 May 26;10:8725. doi: 10.1038/s41598-020-65410-5 (PMC7250915; doi:10.1038/s41598-020-65410-5)

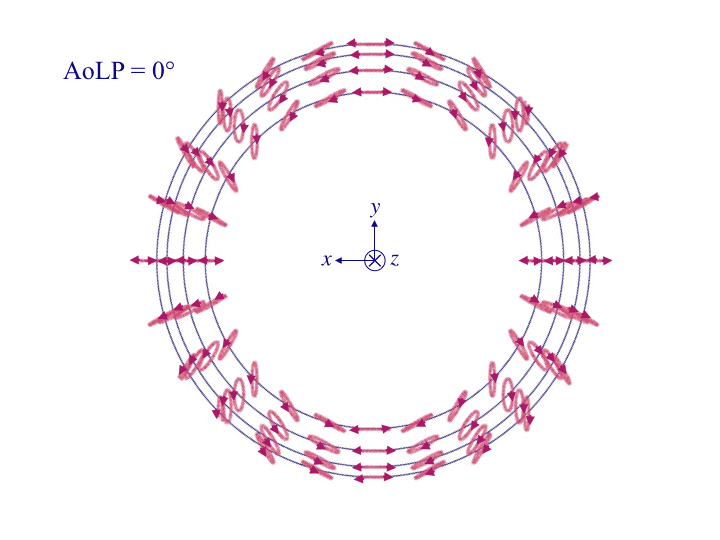

Supplement: Supplementary file 2 — Supplementary Video S1 [file 41598_2020_65410_MOESM2_ESM.gif]
